# Properties and Application of Multifunctional Composite Polypropylene-Based Films Incorporating a Combination of BHT, BHA and Sorbic Acid in Extending Donut Shelf-Life

**DOI:** 10.3390/molecules25215197

**Published:** 2020-11-08

**Authors:** Seyedeh Homa Fasihnia, Seyed Hadi Peighambardoust, Seyed Jamaleddin Peighambardoust, Abdulrasoul Oromiehie, Maral Soltanzadeh, Mirian Pateiro, Jose M. Lorenzo

**Affiliations:** 1 Department of Food Science, College of Agriculture, University of Tabriz, Tabriz 5166616471, Iran; h_fasihnia@tabrizu.ac.ir (S.H.F.); maral.soltanzadeh@gmail.com (M.S.); 2 Faculty of Chemical and Petroleum Engineering, University of Tabriz, Tabriz 5166616471, Iran; j.peighambardoust@tabrizu.ac.ir; 3 Department of Polymer Engineering, Faculty of Engineering, Islamic Azad University, Southern Tehran Branch, Tehran 1584743311, Iran; oromia2000@yahoo.com; 4 Centro Tecnológico de la Carne de Galicia, Rúa Galicia Nº 4, Parque Tecnológico de Galicia, San Cibrao das Viñas, 32900 Ourense, Spain; mirianpateiro@ceteca.net; 5 Área de Tecnología de los Alimentos, Facultad de Ciencias de Ourense, Universidad de Vigo, 32004 Ourense, Spain

**Keywords:** active packaging, migration, sweet snacks, antioxidant, antimicrobial

## Abstract

To extend the shelf-life of packaged donut without the addition of preservative, polypropylene-based active composite films loaded with a combination of sorbic acid, BHA and BHT were prepared by the extrusion moulding method: T_1_ (Control-pure PP-film), T_2_ (PP-BHT1%-SA2%), T_3_ (PP-BHA3%-SA2%) and T_4_ (PP-BHT1%-BHA1%-SA2%). The incorporation of active additives enhanced water vapour permeability (WVP) and increased oxygen permeability of films. Active films had higher antioxidant activity than pure PP in the order T_4_ > T_2_ > T_3_ (89.11, 83.40 and 79.16%). In vitro examinations demonstrated a significant antibacterial effect on *Escherichia coli* and *S. aureus* growth. Overall migration was not significantly different for watery food simulants, while in acidic and fatty foods increased it significantly. The effect of the active films on the fried and packaged donut samples showed significantly higher moisture contents and peroxide values, while acidity was lower. T_2_ film is proposed due to the preservation of the intrinsic properties of the film, increasing the storage period up to 25 to 50 days.

## 1. Introduction

Food packaging allows to protect products against physical, chemical, biological and environmental damage, such as moisture, oxygen, light and microorganisms [1]. Plastic packaging materials such as polyethylene and polypropylene have a great development in food-packaging markets due to their strong barrier properties [2]. In the last two decades, the development of better packaging systems has been promoted, giving rise to the concept of active packaging to prolong storage time, prevent quality deterioration or inhibit the spoilage caused by the growth of microorganisms [3].

Active packaging is one of the effective strategies to minimize the direct use of chemical preservatives in food formulation and it provides the same level of protection, which is achieved using larger quantities of additives, which is directly added to the bulk of food [4,5,6,7]. Incorporating a combination of antioxidant and antimicrobial substances in active packaging films is an emerging packaging system that extends the shelf-life of food products, especially those susceptible to microbial contamination and oxidation deterioration. Flavour or colour changes, microbial spoilage or deterioration and nutritional losses are the main effects caused by oxidation in food [8,9]. To avoid or decline oxidation in food products, different synthetic or natural antioxidants have been included into the packaging film matrix [10,11,12,13,14,15]. Incorporation of synthetic antioxidants such as BHA, BHT and alpha-tocopherol in LDPE/PP films on linoleic acid stability [16,17], lipid oxidation and protein quality of sierra fish muscle during frozen storage [18], and oxidation and odour stability of Asadero cheese [19] has been outlined, demonstrating a promising approach toward prolonging the prepared films’ antioxidant ability in preserving the packed food. Additionally, weak acids such as sorbic acid are used extensively as food-preserving agents, as well as their salts, which could be incorporated with polymeric materials to reduce potential microbial spoilage and extend the shelf-life of food [20]. Furthermore, the combinations of preservatives could have synergistic or antagonistic effects that would be important to elucidate their effect in food complex systems and limit different reactions that lead to food deterioration [21]. 

Donuts (doughnuts), a mixture of flour, oil, egg, sugar, and milk, are fermented and deep fat fried sweet snacks that are very popular and consumed in many countries. An attractive aroma and taste, a golden brown surface, a crisp crust and a porous inner core characterize this product [22,23]. These quick snacks are an important source of energy and nutrients such as fats, carbohydrates and minerals [24]. However, due to their high fat content, off-flavour formation could occur as a result of oxidation reactions [25].

Most studies to date on the quality of the donuts are related to prevention of acrylamide formation during the frying process [26], reduction in oil absorption during the frying process [27], the effects of different process parameters (frying mode, temperature and time) [28], formulation [29] and type and quality of frying oil [30]. However, there are few studies focused on the direct incorporation of plant-based antioxidants in donut formulation to retard acrylamide formation during the frying process [26].

In many food industries, antioxidants, antimicrobials, and antibrowning agents are directly added to the bulk of the food products, which means that their activity is reduced or inhibited due to unwanted interactions with food constituents, and/or during food processing [31,32]. Moreover, in most foods, oxidation and browning reactions, or microbial growth, commonly occur at the surface of the food. Therefore, the addition of active substances via active packaging may be more effective than their addition to the food bulk [2,33]. In this way, continuous replenishment of active substances through a controlled release mechanism from the packaging material to the food surface provides a sustained food protection and the possibility of using a lower amount of additives [34,35,36,37].

Taking into account all the above mentioned points, the aim of this work was to investigate properties of PP-based composite films containing a combination of sorbic acid, BHA and BHT, and evaluate the chemical and microbiological quality of donuts packaged with these active films.

## 2. Results and Discussion

### 2.1. Properties of PP-Based Composite Films

#### 2.1.1. Water Vapour Permeability

Good barrier property against water vapour is an important characteristic of polymers used for packaging of food products. WVP is defined as the quantity of water vapour, which passes through a unit area of polymeric matrix over a defined time. Low WVP values are related to the protective effect of films against water vapour leading to shelf-life extension of packed food [38]. 

**Figure 1:**
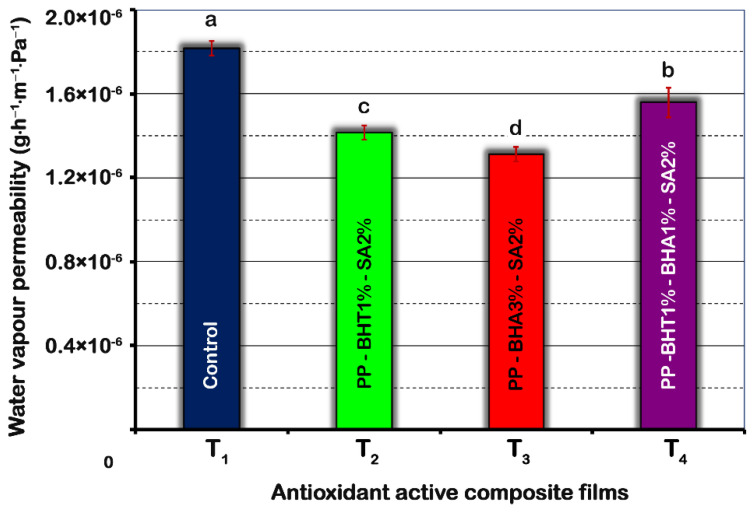
Effect of composition of polypropylene-based films on water vapour permeability (WVP). Means (*n* = 5) with different letters are significantly different (*p* < 0.05). Error bars corresponding to standard deviations.

The WVP of the evaluated active films are shown in Figure 1. All active composite films showed values significantly lower than control (1.82 × 10^−6^ g·h^−1^·m^−1^·Pa^−1^). Among composite films, T_3_ (PP-BHA3%-SA2%) showed the lowest WVP values followed by T_2_ (PP-BHT1%-SA2%) and T_4_ (PP-BHT1%-BHA1%-SA2%) films. Therefore, preventive feature of active composite films against WVP was improved compared to the control by 28%, 23% and 14% for T_3_, T_2_ and T_4_ films, respectively.

As mentioned above, composite film with a higher concentration of BHA (T_3_) showed a lower WVP value. This can be explained by a possible compatibility of antioxidants with the PP matrix, which is an important feature influencing their dispersion, physical and/or chemical interaction with the polymeric matrix. Furthermore, chemical structure and the polarity of filler agents play an important role [39]. The higher polarity of BHA compared to BHT would enhance the interaction of antioxidant functional groups (-OH) with water molecules leading to a compact structure [40], which results in more dense structure with reduced WVP (Figure 1, sample T_3_). A concentration-dependent effect was observed, improving with increasing BHA concentration used. In this way, increasing BHA concentration from 1% to 3% significantly reduced WVP of PP-BHA films.

#### 2.1.2. Antioxidant Properties

**Figure 2:**
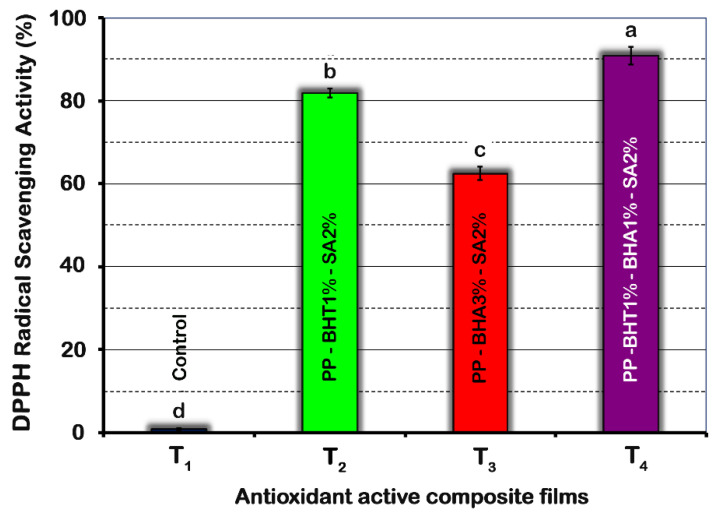
Effect of composition of polypropylene-based films on DPPH radical scavenging activity. Means (*n* = 5) with different letters are significantly different (*p* < 0.05). Error bars corresponding to standard deviations.

Active packaging films with antioxidants delay the oxidation of fats by releasing antioxidants inside the package, allowing the absorption of free radicals [41]. The antioxidant properties of active films are shown in Figure 2. Pure PP film did not display any antioxidant effect, whereas all active composite films had significantly (*p* < 0.05) higher antioxidant activity against 2,2-diphenyl-1-picrylhydrazyl (DPPH) free radicals. Among composite films, antioxidant activity showed the following order: T_4_ (PP-BHT1%-BHA1%-SA2%) > T_2_ (PP-BHT1%-SA2%) > T_3_ (PP-BHA3%-SA2%), with values of 90.8%, 81.8% and 62.5%, respectively. 

The observed difference in antioxidant activity of composite films can be explained by different thermal stability and antioxidant efficiency between the two antioxidants used in this study. BHA is commercially available from 2 and 3 tert-butyl 4-methoxyphenol isomers, since the butyl group has been subjected to ortho- and meta-hydroxyl groups. This steric barrier may cause a lower antioxidant activity of BHA compared to BHT molecule. Furthermore, the tertiary butyl group intervenes in the antioxidant activity of the phenol group, and therefore, is known as a prohibited phenol [42]. This structural barrier in BHA seems to result in its low thermal stability as well [43]. All this together could explain the lower antioxidant activity of composite films with a higher BHA concentration (T_3_ and T_4_ samples).

The highest antioxidant activity of T_4_ sample, which contains the combination of three active substances (2% SA + 1% BHA + 1% BHT), could probably be due to the synergistic effect of BHA and BHT [44]. This behaviour was previously confirmed in other relevant studies. Additionally, sorbic acid acts as a hydrogen donor and inhibits the free radicals in the environment, and it has been identified by the FDA as an authorized food antioxidants [45]. Therefore, the presence of this substance along with the other antioxidants used, especially the combination of the three preservatives (Figure 2, T_4_ sample), boosted the antioxidant properties of the active films.

#### 2.1.3. Antimicrobial Properties

The antimicrobial properties of the active films against *E. coli* and *S. aureus*, as representatives of Gram-negative and Gram-positive bacteria, as well as against *A. niger* are presented in Table 1. All active composite films significantly (*p* ≤ 0.05) reduced the growth rate of Gram-negative and Gram-positive bacteria compared to control. The addition of only 2% sorbic acid in the polypropylene matrix decreased *E. coli* counts from 8.70 to 8.56 (log cfu·mL^−1^) and *S. aureus* from 8.61 to 8.53 (log cfu·mL^−1^) (results not shown). 

According to Table 1, the combination of the same amount of sorbic acid with BHA and BHT was more effective than using SA alone, which is probably due to the antimicrobial properties of the antioxidant additives [21]. Accordingly, the combination of SA with BHT or BHA (T_2_ and T_3_ samples) had 3.5% and 3.7% bacterial inhibition effect on *E. coli*, and 2.4% and 2.7% on *S. aureus*, showing more antibacterial effect on Gram-negative than Gram-positive bacteria [46]. The antimicrobial activity was increased when all three active substances were combined (T_4_ sample) due to the synergistic effect of both antioxidants with each other and with SA. In contrast, although sorbic acid is known as an antimould preservative, the prepared films showed no inhibitory zone in the inoculated *A. niger* cultures. This could possibly be explained by the lower thickness (0.026 mm) of the film samples, which did not spread enough SA into the mould culture.

#### 2.1.4. Migration Test

Numerous substances including different food additives, which are intentionally added into active packaging materials, can have direct contact with food leading to different degrees of migration into food [47]. Migration extent could be affected by various factors including food nature, packaging materials, temperature and time of contact, characteristics of migrating additives and content of potential migrants in packaging materials [48]. Considering a broad type of food systems, different food analogues are used to perform migration tests in laboratories. Hence, three food simulants, distilled water, acetic acid in water (3% *w*/*v*) and ethanol in water (95% *v*/*v*) are considered as food simulants for aqueous foods (pH > 4.5), acidic foods (pH ≤ 4.5) and fatty foods, respectively.

Table 2 shows overall migration of active composite films compared to control in the food simulants mentioned. For all simulants used, composite active films showed significantly (*p* < 0.05) higher overall migration than control. There was no significant (*p* > 0.05) difference in migration of composite films with different additives in distilled water. However, both acetic acid and ethanol media significantly increased the overall migration of all active composite films. Nevertheless, they did not exceed the threshold of 10 mg.dm^−2^ as allowed by EN 1186-1 standards [49].

The observed differences depend on the intrinsic properties of each additive such as molecular weight and polarity, which will influence their mobility along the polymer chains [50]. The overall migration of additives in the three active films was higher in fatty food simulant than in the other two simulants, which is due to the high polarity (solubility) and hydrophobicity of additives (BHT > BHA > SA) in 95% (*v*/*v*) ethanol [1,51].

#### 2.1.5. Oxygen Permeability

**Figure 3:**
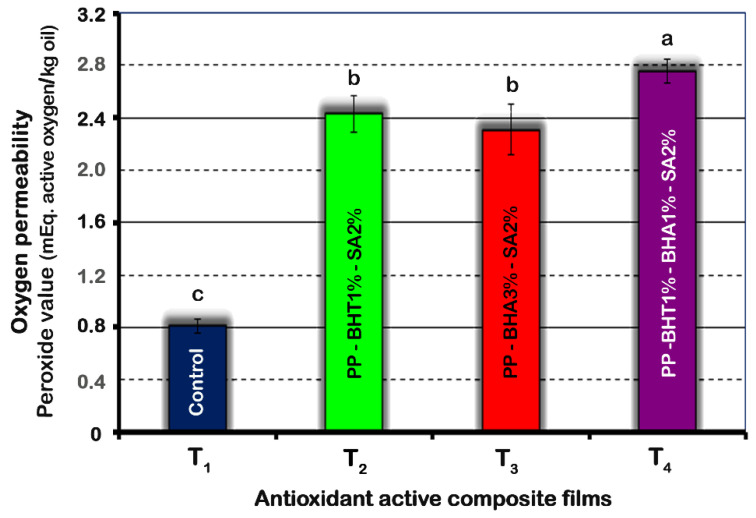
Effect of composition of polypropylene-based films on oxygen permeability of composite films. Means (*n* = 3) with different letters are significantly different (*p* < 0.05). Error bars corresponding to standard deviations.

The barrier properties of the packaging material are dependent on food environment conditions—i.e., temperature and RH. The diffusion of additives across the film is determined by film thickness, surface area, and permeability of film against specific gases or vapour at a given temperature [52]. The oxygen permeability of composite films is shown in Figure 3. 

Addition of antioxidants and antimicrobial agents to the PP-polymer matrix in all samples significantly (*p* < 0.05) increased their oxygen permeability. Thus, peroxide values raised by about 3-fold for composite polymers incorporating additives compared to the pure PP film (*p* ≤ 0.05). The gas permeability of composite films, in particular oxygen, depends on factors such as molecular weight, molecular volume, hydrophobicity and the degree of polymer chain mobility. Therefore, additives with more hydrophobic properties result in less oxygen solubility in the films, leading to an increased in oxygen permeability [15]. The higher oxygen permeability values of the films containing BHT (T_2_ and T_4_) could be explained taking into account the hydrophobicity order of the additives as BHT > BHA > SA. 

### 2.2. Chemical and Microbiological Quality of Donuts during Shelf-Life

#### 2.2.1. Donut Moisture Content

The results of moisture measurement of donut samples are shown in Table 3. The statistical analysis showed a significant effect of active films, storage time and the interaction of these two factors on donuts moisture content. According to F-values, the effect of storage time was higher than the effect of the type of films (187.5 vs. 32.6). 

Donut moisture content after frying was 11.6%, which was significantly reduced to 1.87% after 75 days of storage for samples packed in the pure PP film (T_1_). Our results are consistent with those reported by other authors [53]. To a lesser extent, this reduction in moisture content was also observed in donuts packed in T_2_, T_3_ and T_4_ films (Table 3). All active films were significantly (*p* < 0.05) able to retain higher moisture content in donuts at all tested storage intervals. However, there was no significant difference among active films in retaining moisture content at each time interval. 

The results of moisture content were linked to those found for WVP (Figure 1). Decreasing the permeability of active films to water vapour, especially for T_2_ and T_3_ samples, could cause the moisture to be retained inside the package. This fact could explain the higher moisture content of donuts packed in T_2_ and T_3_ films. 

#### 2.2.2. Donut Peroxide Value

Donut has a fat content of about 10–25%, which varies depending on the formulation and the frying conditions [26]. Foodstuffs contain high amounts of unsaturated fatty acids, so they are highly susceptible to oxidation. The changes occurring during fat oxidation lead to a reduction in nutritional value [54]. The lipid oxidation is accelerated significantly at high temperatures and depends strongly on oxygen availability. Peroxide value (PV) is most commonly used as indicator of the early stages of oxidation in fats and oils [14].

PV values of fat extracted from fresh and packed donuts in different PP-based active films during storage time are shown in Table 3. There was a significant effect of type of active film, storage time and the interaction of these two factors on PV values (*p* ≤ 0.05), in which the effect of storage time was more pronounced than the effect of the type of packaging film. PV for fried unpacked samples was 0.45 mEq/kg. The results showed that there were no significant differences among films stored for 25 days. On day 50, T_2_ and T_4_ films (containing BHT) showed lower PVs than T_3_ films (incorporating BHA). Therefore, it seems that BHT is more effective in lowering PV compared to other antioxidants. This is in agreement with the results found by Torres-Arreola et al. [14] and Jongjareonrak et al. [40]. After storage for 75 days, PVs were significantly increased for all packages and contrary to expectations, all active films showed significantly higher PVs than control. Increase in PV over storage time was due to the progression of oxidation reactions during storage. However, the maximum PVs reached were within the acceptable level (3 mEq/kg oil).

#### 2.2.3. Donut Acidity

Results of free fatty acid (FFA) contents of fresh and packed donuts in different PP-based active films during storage are shown in Table 3. The FFA content of fresh donut was 0.20% (oleic acid-based). The FFA content of donuts packed in pure PP film was significantly (*p* < 0.05) increased after storage up to 0.25%. Moreover, donuts packed in active films had significantly lower values than control at all storage times. There was no significant difference between active films on FFA content of donuts (*p* > 0.05). However, there was a gradual non-significant reduction (from 0.16 to 0.09%) in all active films during storage. This reduction in FFA content of packed donuts was consistent with findings of Torres-Arreola et al. [14]. Similar to peroxide value results, FFA values were lower than maximum acceptable limits (1 wt% based on oleic acid).

#### 2.2.4. Microbial Shelf-Life

**Figure 4:**
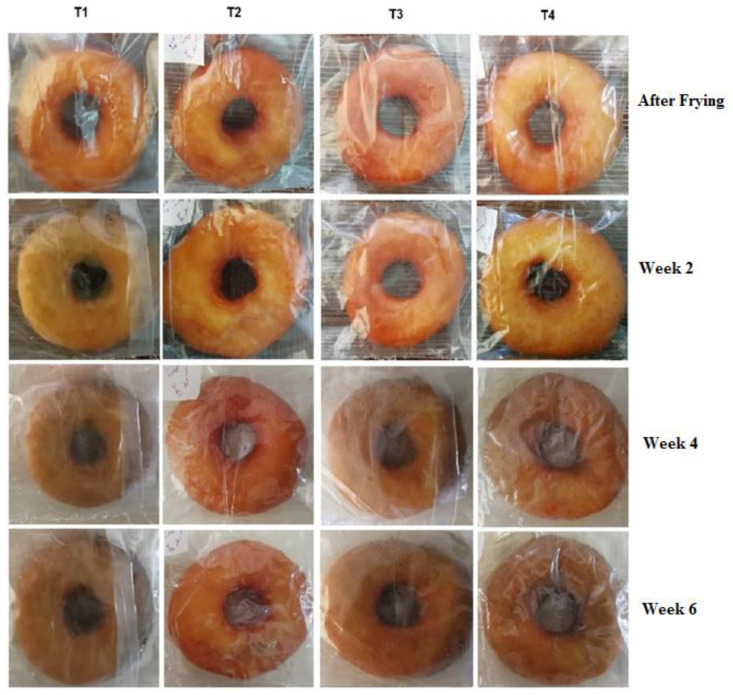
Effect of composition of polypropylene-based films on fungal growth during storage time of packaged donuts.

The microbiological shelf-life of donuts packed in active films were evaluated by the observation of mould colonies on their surface upon storage at room temperature. To accelerate the mould growth, the surface of donuts was smeared with 0.1 mL *A. niger*. As shown in Figure 4, there was no sign of mould growth in any of the samples at week 2. However, at the end of the second week, early signs of fungal colonies were observed in donut packed in control film (T_1_). 

At the end of the week 4, mould growth was increased in all samples except donuts packed in T_2_ film (containing 2% SA and 1% BHT). At the end of week 6, obvious signs of fungal growth were seen at the surface of samples packed in T_1_, T_3_ and T_4_ films, observing a green layer as a result of the growth of fungal mycelium. This effect was less pronounced for donuts packed in T_2_ film after 6 weeks (Figure 4). As indicated above, the control sample did not show any inhibitory effect on *A. niger*, while the addition of sorbic acid along with BHA and BHT to the PP polymeric matrix produced an inhibitory effect. The greatest antifungal effect of active films during 6 weeks was notice to T_2_ film followed by T_4_ sample. Although T_3_ film showed less antimould effect compared to other films, it allowed an increase in donut shelf-life up to two weeks compared to the control film. Inhibition of *A. niger* growth by polymer composites containing sorbic acid has been confirmed by various studies [55].

The antimicrobial properties of the films are explained by the migration of the incorporated active agents, being the large molecular structure one of the main requirements for attaching to the polymer matrix and maintaining its function on the cell membrane of the target microorganism. These materials are likely to bind to enzymes or microbial proteins, and without migration, effectively inhibit surface growth of microorganisms [18,56]. According to Dutta et al. [57], the hydrophilic–hydrophobic balance of a substance, as well as the nature of long chains (hydrophobic), is more consistent with the cytoplasmic lipid membrane, which is largely responsible for its antimicrobial properties. Therefore, the release of the antimicrobial agent through the cell wall requires optimum hydrophobicity.

Overall, the findings obtained were confirmed by those found by Silveira et al. [58], who established the use of antimicrobial films, especially those containing higher concentrations of sorbic acid, which could be more effective than the direct addition of antimicrobial agents to food since the antimicrobial agent migrates gradually throughout the storage. Thus, its concentration should be considered in such amount, which could apply its antimicrobial properties over time and prevent or delay microbial degradation, in view of the fact that microbial contamination at the surface of fresh or processed foodstuffs is more intense and requires active control over the surface growth of microorganisms.

## 3. Materials and Methods 

### 3.1. Active Film Preparation

The active films were made by mixing PP granules (Moplen Z30S: melt flow index of 25 dg·min^−1^, and density of 0.9 g·cm^−3^ suitable for food contact) (Marun Petrochemical Co., Bandar-e-Emam, Iran), antioxidants (Butylated hydroxytoluene; BHT and Butylated hydroxyanisole; BHA) (Merck, Darmstadt, Germany) and an antimicrobial additive (sorbic acid; SA) (AppliChem GMbH, Darmstadt, Germany) at concentrations shown in Table 4: T_1_ (Control-pure PP-film), T_2_ (PP-BHT1%-SA2%), T_3_ (PP-BHA3%-SA2%) and T_4_ (PP-BHT1%-BHA1%-SA2%). 

The mixture of materials was introduced into a co-rotating twin-screw extruder (SM PLATEK, Ansan-si, Korea) with screw diameter of 20 mm, L/D ratio of 32, screw speed of 145 rpm and a barrel temperature of 145–200 °C. The molten extrudate (cylindrical shape) was left the die and passed through a cold water basin before being cut into granules. These granules were transferred to a single-screw blowing extruder (screw diameter of 20 mm; L/D ratio of 26) (Ghioldi Srl., Marnate, Italy). The screw speed and temperature of barrel sections were 145 rpm and 160–190 °C, respectively to produce packaging films with a width and thickness of 100 and 0.026 mm, respectively [2]. Then, the films were cut into 100 × 100 mm pieces for packaging of donut samples.

### 3.2. Determination of Film Properties

#### 3.2.1. Water Vapour Permeability (WVP)

A gravimetric ASTM E96-16 method [59] was applied to evaluate water permeability of films according to González Sandoval et al. [60] with few modifications. Conditioned film discs (Ø = 16 mm, 55% relative humidity, RH) were sealed to glass vials of 45 mm height with a hole of 8 mm on the cap containing anhydrous CaSO_4_ (0% RH), and the vials were weighed and transferred into a desiccator maintained at 97% RH with a saturated salt solution of potassium sulphate at 25 °C. The weight gain of the vials was measured periodically over a total time of 96 h, which indicated the amount of water vapour transferred from the films or water vapour transmission rate (WVTR). WVP was then calculated as follows:

$$\mathrm{WVP}=\frac{\mathrm{WVTR}}{A}\frac{X}{\Delta P_{v}}$$

where WVP is the water vapour permeability (g·m^−1^·h^−1^·Pa^−1^); WVTR is the water vapour transmission rate (g·h^−1^), which was calculated by plotting film weight gain data over time (h) through a linear regression (R^2^ > 0.98); X is the mean of film thickness (m); A is the area of WVT (m^2^); and ∆Pv is the vapour pressure difference (3073.9 Pa) between the atmosphere of two sides of the films; inside the vial with anhydrous CaSO_4_ with RH = 0% and the desiccator containing saturated K_2_SO_4_ solution with RH = 97%. Due to the presence of anhydrous CaSO_4_, vapour pressure inside the vial was considered zero. In specific terms, WVTR was considered as the slope of curve plotting the final film weight (weight gain at the moment of measurement) minus the initial weight of the sample (W_f_−W_0_) over the time. Three replicates were conducted for the same treatment.

#### 3.2.2. Antioxidant Properties

There are in situ methods to determine antioxidant activity in packaging films [61]; however, 1,1-Diphenyl-2-Picrylhydrazyl (DPPH) radical scavenging activity (RSA) is by far the most commonly used method for the evaluation of the antioxidant activity of composite films. For this purpose, films were cut into small pieces (0.1 g) and transferred in a Falcon tubes. Methanol (2 mL) was added to each sample, mixed for 60 s using a shaker (Velp Scientifica Srl., Usmate, Italy) and stored at room temperature for 2 h. Then, DPPH solution in methanol (2 mL 0.1 mM) was added to methanol extract (500 μL). The mixture was blended vigorously and kept for 30 min at room temperature in the dark. Absorbance of the mixture was measured at 517 nm using a spectrophotometer (UNICO, UV-2100, Dayton, NJ, USA). Control was prepared using the same procedure, but methanol was used instead of the sample extract. DPPH radical scavenging activity (%) was calculated as follows [12]:

$$\mathrm{RSA}\;\left(\%\right)=\left(1-\frac{A_{\mathrm{sample}}}{A_{\mathrm{control}}}\right)\times 100$$

where A_sample_ is absorbance of film extract, and A_control_ is absorbance of control.

#### 3.2.3. In Vitro Antimicrobial Properties

In vitro antimicrobial activity of polypropylene-based composite films was evaluated against *Escherichia coli* (ATCC 25922), *Staphylococcus aureus* (ATCC 29523) and *Aspergillus niger* (ATCC 9029). For bacterial tests, circle film specimens with diameter of 20 mm were located in two separate sterile falcons containing 10 cm^3^ of each bacteria of seeding culture and two others without microorganisms (as control). Falcons containing film samples and microorganisms were incubated for 24 h at 37 °C, then the absorption of the solutions was measured at 625 nm using a spectrophotometer (UNICO, UV-2100, Dayton, NJ, USA). Antifungal properties of active films were tested against *A. niger* using an inhibitory zone test on a potato dextrose agar (PDA) semisolid medium (QUELAB, Montreal, QC, Canada). For this purpose, films samples were sited into PDA containing petri dishes, where an amount of 0.1 mL seeding culture was previously spread out. The petri dishes were then incubated at 25 °C for 96 h. The diameter of inhibition zones was measured in three different places and an average was reported [36]. 

#### 3.2.4. Migration Test

Overall migration was determined according to British Standard EN 1186-1 [49]. In this procedure, distilled water, 3% acetic acid in water and 95% ethanol in water were considered as food simulants for aqueous foods (pH > 4.5), acidic foods (pH ≤ 4.5) and fatty foods, respectively. To measure the migration rate, film strips (25 × 20 mm) were cut and immersed completely in simulants. Migration tests were performed inside glass tubes, which were covered with parafilm and incubated in an oven (Memmert GmbH, Schwabach, Germany) at 40 ± 1 °C for 10 days. During testing, simulant evaporation was compensated by keeping the solvent at the same initial level. Finally, simulants were evacuated into small glass cells and dried at 105 °C to a constant mass. The overall migration (mg/dm^2^) of films was calculated by the following equation [48]:

$$\mathrm{Overal}\;\mathrm{migration}=\frac{M_{2}-M_{1}}{A}$$

where M_2_ refers to glass cell weight (mg) after simulant evaporation, M_1_ is empty glass cell weight (mg) and A is film specimen surface area (dm^2^). Measurements were performed in triplicates for each master batch film at different locations of prepared films. 

#### 3.2.5. Oxygen Permeability

The permeability of PP-based composite films to oxygen was determined indirectly. The peroxides and hydroperoxides formed during the initial stages of lipid peroxidation can be measured by determining the peroxide value (PV). Thus, higher PVs correspond to higher oxygen permeability (OP) of the films. To determine OP, film discs (Ø = 16 mm) were first conditioned (55% relative humidity, RH for 24 h) and sealed in glass vials (Ø = 8 mm, H = 45 mm) containing 10 mL of pure oleic acid. The vials were then incubated for 20 days in conditions of 55% RH and at 25 ± 1 °C. PV of oleic acid expressed as mEq oxygen/kg oil was measured according to the AOAC method 965.33 [62]. PVs were considered as the oxygen permeability of the tested films [63]. Measurements were performed in triplicates for each master batch film at different locations of prepared films.

### 3.3. Donut Preparation

The formulation for donut preparation is shown in Table 5. At first, dry instant yeast (Dr. Oetker Co., İzmir, Turkey) was activated by adding icing sugar (2 g), wheat flour (15 g) and water (40 g). Wheat flour (Athar-bonab Co., Bonab, Iran; with moisture, crude protein, ash and wet gluten contents of 13.1%, 12.6%, 0.43%, 29.4%, respectively) was mixed with iodine-free sodium chloride (Sepid-Daneh Co., Shiraz, Iran) and sieved. It was then mixed with butter in a spiral-type vertical mixer (Sepah-Kar Co., Isfahan, Iran) at a speed of 60 rpm for 10 min at room temperature (24 °C). Activated yeast suspension together with other ingredients (Table 5) was added to the mixture, and the mixing was continued for 10 min. First proofing (T = 35 °C, t = 120 min, RH = 70%) was carried out, and the dough was then manually kneaded for 5 min. The leavened dough was rolled out to an approximately 10 mm sheet and cut with a donut cutter-mould with an inner and outer diameter of 20 and 70 mm, respectively. Final proofing (T = 37 °C, t = 15 min, RH = 90%) was then applied on the moulded doughs [64]. Dough pieces were deep fat fried for 4 min in a kitchen-type fryer (Moulinex, Écully, France), filled with 3 L sunflower oil set to a constant temperature of 180 °C. Fried donuts were let to cool down to ambient temperature on a paper towel for 40 min to remove the excess oil from their surface. Fried donuts were packed with active antioxidant films mentioned in Table 4 and stored at room temperature for 75 days. A control donut was considered without packaging for subsequent tests. The analyses were carried out for packaged samples during the storage period at 25-day intervals.

### 3.4. Donut Chemical Analyses

#### 3.4.1. Moisture Measurement

The quality of some food products, such as dried fruits and snacks, is reduced by increasing the moisture content, while the quality of some bakery products such as cakes and donuts is negatively affected by reducing their moisture. The moisture content of donuts during the storage period plays a significant role in maintaining its sensory properties and in controlling the microbial spoilage. The moisture content of donuts packed in active composite films was examined during the 75 days of storage. The control sample was considered as fresh fried donut with no packaging. Moisture content of samples was determined based on weight loss after drying at 105 °C for 3 h in an oven [65].

#### 3.4.2. Fat Extraction

Donut fat extraction was carried out by the cold extraction method according to Lim et al. [66]. Samples were weighed and mixed with solvent (hexane) at a ratio of 1:3 (*w*/*v*) followed by gentle shaking for 12 h. The resulting mixture was then filtered using filter paper, and fat extracted by a vacuum evaporator (HAHN SHIN SCIENTIFIC CO., Bucheon-si, Korea) equipped with a water bath at 55 to 60 °C. The extracted fat was used to measure fat acidity and peroxide values.

#### 3.4.3. Peroxide Value 

Peroxide value was measured according to the AOAC 965.33 method [62]. Oil samples (4 g) were mixed with 30 mL of peroxide solution. This solution was prepared from chloroform (Merck, Darmstadt, Germany) and acetic acid (Merck, Darmstadt, Germany) in a ratio of 2:3 (*v*/*v*). Saturated potassium iodide solution (Merck, Darmstadt, Germany) (0.5 mL) was then added. After 1 min of storage in dark conditions, 30 mL of distilled water and a few droplets of 1% starch solution were added to make a blue colour if peroxide appeared in the sample. Titration was performed by sodium thiosulphate 0.1 N (Merck, Darmstadt, Germany) to disappear the blue colour. Peroxide values were calculated according to the following equation:

$$\mathrm{Peroxide}\;\mathrm{value}\;\left(\frac{\mathrm{mEq}.\mathrm{Active}\;\mathrm{Oxygen}}{\mathrm{kg}\;\mathrm{Oil}}\right)=\frac{\left(100\times N\times V\right)}{W}\times 100$$

where N is the sodium thiosulfate normality, V is the sodium thiosulfate volume (mL) and W is the fat weight (kg).

#### 3.4.4. Fat Acidity (Free Fatty Acids) 

Free fatty acid (FFA) content of fat was determined according to the AOAC 965.33 method [62]. An oil sample (2 g) was mixed with 30 mL of warm neutralized alcohol including 2 mL of phenolphthalein reagent. The mixture was titrated with sodium hydroxide solution 0.1 N (Merck, Darmstadt, Germany) until a constant white pink–colour appeared. The amount of FFA was determined using the following equation:

$$\mathrm{Fat}\;\mathrm{acidity}\;\left(\mathrm{FFA}\right)\;\mathrm{based}\;\mathrm{on}\;\mathrm{oleic}\;\mathrm{acid}=\frac{\left(M\times N\times V\right)}{W}$$

where V is the volume (mL) of consumed sodium hydroxide for titration, W is oil weight (g), M is acid molarity (for oleic acid = 28.2 g/mol) and N is sodium hydroxide normality (0.25 N).

### 3.5. In-Vivo Antimicrobial Properties of Active Films

The appearance and duration of emerging moulds grown at the surface of donuts was used as in vivo antimicrobial test to evaluate the shelf-life of donuts packaged in different active films. High fat content of fried donuts retarded mould growth on the surface of the product. Thus, to accelerate mould growth rate and to evaluate antifungal activity of the packaging films, the surface of donuts was inoculated with 0.1 mL of *Aspergillus niger*. Donuts were then packed and stored in similar conditions to the non-inoculated control samples. The growth of mould was visually monitored and recorded during storage days [55].

### 3.6. Statistical Analyses

Statistical analysis was performed based on a completely randomized design (CRD) using Minitab 16 software (ver. 16, State college, PA, USA). Normal distribution and homogeneity of variance were previously tested using Minitab 16 software (ver. 16, State college, PA, USA). One-way ANOVA and multiple range comparison Tukey’s tests were used to determine the significant (*p* < 0.05) difference between the means.

## 4. Conclusions

All three types of active films exhibited favourable antioxidant and antimicrobial properties, which, based on the thermal, mechanical and protective properties observed, can be used in various applications. Taking into account the main purpose of this study, to increase the shelf-life of packaged donut in active films, the T_2_ film sample, containing 2% sorbic acid and 1% BHT, is proposed for further uses due to preserving the intrinsic properties of the film and applying the most antimicrobial effect to increase the storage period of donuts up to a minimum of 25 and a maximum of 50 days.

## Figures and Tables

**Table 1:** The results of the antimicrobial properties of PP-based active films.

	Film Samples	*E. coli* (Log cfu·mL^−1^)	*S. aureus* (Log cfu·mL^−1^)	*A. niger* (Inhibition Area)
T_1_	Control	8.70 ± 0.02 ^a^	8.61 ± 0.00 ^a^	0
T_2_	PP-BHT1%-SA2%	8.41 ± 0.01 ^b^	8.40 ± 0.01 ^b^	0
T_3_	PP-BHA3%-SA2%	8.38 ± 0.01 ^bc^	8.38 ± 0.00 ^b^	0
T_4_	PP-BHT1%-BHA1%-SA2%	8.35 ± 0.01 ^c^	8.20 ± 0.01 ^c^	0

Data are mean (*n* = 3) ± standard deviations. Means with different letters in each column are significantly different (*p* < 0.05).

**Table 2:** The results of overall migration (mg/dm^2^) in different food simulants.

	Film Samples	Aqueous Foods (pH > 4.5)	Acidic Foods (pH ≤ 4.5)	Fatty Foods
T_1_	Control (pure PP-film)	1.32 ± 0.37 ^bA^	1.32 ± 0.37 ^bA^	1.66 ± 0.35 ^bA^
T_2_	PP-BHT1%-SA2%	2.32 ± 0.39 ^aB^	2.12 ± 0.63 ^abB^	6.07 ± 0.40 ^aA^
T_3_	PP-BHA3%-SA2%	2.84 ± 0.44 ^aB^	3.66 ± 0.56 ^aB^	7.82 ± 0.97 ^aA^
T_4_	PP-BHT1%-BHA1%-SA2%	2.43 ± 0.50 ^aC^	3.96 ± 0.48 ^aB^	6.64 ± 0.56 ^aA^

Data are mean (*n* = 3) ± standard deviations. Means with different letters are significantly different (*p* < 0.05). ^A–C^ Different capital letters indicate significant (*p* < 0.05) differences among different food simulants. ^a–b^ Different lowercase letters indicate significant (*p* < 0.05) differences among film samples.

**Table 3:** Moisture content, peroxide value and free fatty acid contents of fresh and packed donuts in different PP-based active films during storage time.

Analysis	Treatments	Storage Time			
		Day 1	Day 25	Day 50	Day 75
Moisture (%)	Fresh donut (not packed)	11.6 ± 0.52	-	-	-
	Control (pure PP) film (T_1_)	-	5.48 ± 0.18 ^Ab^	4.30 ± 0.06 ^Bb^	1.87 ± 0.81 ^Cb^
	PP-BHT1%-SA2% film (T_2_)	-	7.00 ± 0.37 ^Aa^	6.23 ± 0.55 ^Aa^	4.61 ± 0.34 ^Ba^
	PP-BHA3%-SA2% film (T_3_)	-	7.15 ± 0.85 ^Aa^	6.07 ± 0.21 ^Aa^	4.53 ± 0.21 ^Ba^
	PP-BHT1%-BHA1%-SA2% film (T_4_)	-	6.81 ± 0.03 ^Aa^	3.26 ± 0.18 ^Bc^	3.21 ± 0.10 ^Bb^
Peroxide value (mEq. active O_2_/kg oil)	Fresh donut (not packed)	0.45 ± 0.04	-	-	-
	Control (pure PP) film (T_1_)	-	0.42 ± 0.11 ^Ca^	0.68 ± 0.07 ^Bc^	1.62 ± 0.40 ^Ab^
	PP-BHT1%-SA2% film (T_2_)	-	0.43 ± 0.07 ^Ca^	1.94 ± 0.15 ^Bab^	2.80 ± 0.25 ^Aa^
	PP-BHA3%-SA2% film (T_3_)	-	0.39 ± 0.11 ^Ba^	2.39 ± 0.29 ^Aa^	2.60 ± 0.27 ^Aa^
	PP-BHT1%-BHA1%-SA2% film (T_4_)	-	0.49 ± 0.13 ^Ca^	1.75 ± 0.21 ^Bb^	2.60 ± 0.24 ^Aa^
Free fatty acids (wt%, oleic acid-based)	Fresh donut (not packed)	0.20 ± 0.02	-	-	-
	Control (pure PP) film (T_1_)	-	0.19 ± 0.03 ^Ba^	0.22 ± 0.02 ^Ba^	0.25 ± 0.03 ^Aa^
	PP-BHT1%-SA2% film (T_2_)	-	0.16 ± 0.02 ^Ab^	0.15 ± 0.02 ^Ab^	0.12 ± 0.03 ^Ab^
	PP-BHA3%-SA2% film (T_3_)	-	0.15 ± 0.02 ^Ab^	0.13 ± 0.03 ^Ab^	0.09 ± 0.02 ^Ab^
	PP-BHT1%-BHA1%-SA2% film (T_4_)	-	0.16 ± 0.02 ^Ab^	0.12 ± 0.01 ^Bb^	0.11 ± 0.02 ^Bb^

Data are mean (*n* = 3) ± standard deviations. ^A–C^ Different capital letters indicate significant (*p* ≤ 0.05) differences among storage times. ^a–b^ Different lowercase letters indicate significant (*p* ≤ 0.05) differences among film samples.

**Table 4:** Concentrations of antioxidants and antimicrobial agents part “Per Hundred Rubber” (PHR) in the formulation of PP-based films.

	Film Samples	PP	BHA	BHT	SA
T_1_	Control	100	-	-	-
T_2_	PP-BHT1%-SA2%	97	-	1	2
T_3_	PP-BHA3%-SA2%	95	3	-	2
T_4_	PP-BHT1%-BHA1%-SA2%	96	1	1	2

**Table 5:** Formulation of donut samples.

Ingredients	Amount (g)	Amount (%)
Wheat flour	670	53
Water	57	4.5
Milk	210	16.5
Sugar	55	4.5
Whole egg	170	13
butter	100	8.0
Instant yeast	5.5	0.5
